# Compatibility in the *Ustilago maydis*–Maize Interaction Requires Inhibition of Host Cysteine Proteases by the Fungal Effector Pit2

**DOI:** 10.1371/journal.ppat.1003177

**Published:** 2013-02-14

**Authors:** André N. Mueller, Sebastian Ziemann, Steffi Treitschke, Daniela Aßmann, Gunther Doehlemann

**Affiliations:** Max Planck Institute for Terrestrial Microbiology, Marburg, Germany; Purdue University, United States of America

## Abstract

The basidiomycete *Ustilago maydis* causes smut disease in maize, with large plant tumors being formed as the most prominent disease symptoms. During all steps of infection, *U. maydis* depends on a biotrophic interaction, which requires an efficient suppression of plant immunity. In a previous study, we identified the secreted effector protein Pit2, which is essential for maintenance of biotrophy and induction of tumors. Deletion mutants for *pit2* successfully penetrate host cells but elicit various defense responses, which stops further fungal proliferation. We now show that Pit2 functions as an inhibitor of a set of apoplastic maize cysteine proteases, whose activity is directly linked with salicylic-acid-associated plant defenses. Consequently, protease inhibition by Pit2 is required for *U. maydis* virulence. Sequence comparisons with Pit2 orthologs from related smut fungi identified a conserved sequence motif. Mutation of this sequence caused loss of Pit2 function. Consequently, expression of the mutated protein in *U. maydis* could not restore virulence of the *pit2* deletion mutant, indicating that the protease inhibition by Pit2 is essential for fungal virulence. Moreover, synthetic peptides of the conserved sequence motif showed full activity as protease inhibitor, which identifies this domain as a new, minimal protease inhibitor domain in plant-pathogenic fungi.

## Author Summary

Biotrophic plant-pathogen interactions depend on the suppression of the host immune system. In the compatible *Ustilago maydis*–maize interaction, living host cells are colonized without inducing defense responses. Thereby, host metabolism and gene expression are widely reprogrammed, resulting in the formation of large plant tumors. This manipulation of the host plant requires so-called effector proteins, which are secreted by *U. maydis* to promote disease. Recently we identified the effector Pit2 (protein involved in tumors 2), which is encoded within a cluster of four plant-induced genes. Deletion mutants for *pit2* can infect maize plants but fail to maintain biotrophy. They elicit various defense responses and fail to induce tumors. We now show that Pit2 interacts with a group of apoplastic maize cysteine proteases, which themselves are crucial factors for maize defense induction. Pit2 efficiently inhibits these proteases to suppress maize host resistance. By sequence comparisons, we identified a 14 amino acid motif of Pit2, which is required for protease inhibition and, consequently, for *U. maydis* virulence. Moreover, synthetic peptides of this motif, but not mutated versions, inhibit maize cysteine proteases. This identifies a novel type of fungal protease inhibitor with an essential role in suppression of maize immunity.

## Introduction

To successfully infect their hosts, plant pathogens secrete a highly diverse continuum of molecules that interfere with a wide range of physiological processes in the infected host. Such molecules are commonly termed effectors. A growing number of microbial effector proteins that contribute to virulence are described in recent literature. Bacterial pathogens such as *Xanthomonas* species inject proteins to the host cytoplasm via a specialized type-III secretion apparatus to suppress host immunity and manipulate gene expression in the infected cells [1], [2]. Filamentous fungi and oomycetes also secrete effectors, which are either translocated to the host cytoplasm or act in the plant apoplast [3]–[5]. While mechanisms of effector uptake into host cells are controversially discussed, a growing body of evidence shows crucial roles in suppression of host immunity by apoplastic space effectors that mainly act as inhibitors of plant enzymes. The oomycete pathogen *Phytophthora infestans* produces several apoplastic enzyme inhibitors, such as the GIPs (glucanase inhibitor proteins) that inhibit plant endo-beta-1,3-glucanases [6]. Kazal-like protease inhibitors of *P. infestans* were found to interact with the apoplastic serine protease P69B in tomato [7], [8]. In addition, two cystatin-like proteins interfere with the tomato cysteine proteases PIP1 and RCR3 [9], [10].

In the fungal tomato pathogen *Cladosporium fulvum*, a set of apoplastic effectors has been identified as avirulence products [11]. One of these proteins, Avr2, does interact with tomato cysteine proteases including RCR3 and PIP1 [12]–[14], pointing to a central role of these proteases as common effector targets [9]. Consequently, expression of Avr2 is also required for full virulence of *C. fulvum* in tomato [12]. So far, seven effector proteins secreted by the tomato wilt pathogen *Fusarium oxysorum* f. sp. *lycopersici* were found in the xyleme sap of infected plants [15]. Although their actual molecular functions still remain unknown, these “secreted in xylem” (Six) proteins are linked with fungal virulence and also comprise avirulence (Avr) proteins that are recognized by their cognate receptors in resistant tomato plants [16], [17]. This is a situation different to *C. fulvum*, where apoplastic effectors are required to suppress basal resistance in susceptible plants and at the same time act as avirulence products in resistant host cultivars [9].

For the biotrophic interaction of the basidiomycetous fungal pathogen *Ustilago maydis* and its host plant maize, no avirulence factors and cognate resistance genes are known [18]. The *U. maydis* genome encodes 386 putative effector proteins, which to a large extent are encoded by gene clusters that are specifically expressed during plant colonization [19]. Deletion mutants for such gene clusters show various pathogenicity phenotypes reaching from hypervirulence to complete arrest of infection, which can be observed at different stages of interaction as well as depending on the infected plant organ [20], [21]. Nevertheless, for most of the *U. maydis* effectors, only little is known on their subcellular localization and molecular functions. Recent progress showed a secreted chorismate mutase, which is translocated from intracellular *U. may*dis hyphae to the plant cytoplasm, where it interferes with salicylic acid (SA) synthesis [22]. An apoplastic core effector with an essential function for *U. maydis* virulence is Pep1. Deletion mutants for *pep1* are arrested during epidermal penetration and induce various defense responses [23]. The protein accumulates in biotrophic interface around intracelluar biotrophic hyphae and at sites of cell-penetrations where it blocks the oxidative burst via a direct inhibition of host peroxidases [24], giving a first example for a fungal effector directly interfering with the ROS-generating system of the host plant.

Another central component of apoplastic immunity in maize are papain-like cysteine proteases, which are activated by SA and, in turn, by themselves trigger the expression of *PR*-genes and host cell death [25]. Establishment of biotrophy in the maize - *U. maydis* interaction requires the suppression of these proteases by an endogenous maize cystatin (CC9), which is transcriptionally induced during epidermal penetration in the compatible interaction [25]. This is also in line with the observation made in the *P. infestans* - tomato interaction, where expression of the extracellular papain-like tomato protease C14 contributes to host immunity [26]. Here, the *P. infestans* effector AVRblb2 was shown to promote virulence by preventing secretion of the protease to the apoplast [26].

In a recent study we identified the *U. maydis* effector Pit2, which is situated in a cluster of four plant-induced genes [27]. Pit2 is secreted to the biotrophic interface and spreads in the apoplastic space around colonized maize cells. Deletion mutants for *pit2* are able to establish biotrophy, but only few days after infection the host colonization is blocked, which coincides with a broad induction of plant defense and collapse of infected host cells [27].

In this study, we present the functional characterization of Pit2 and demonstrate that it acts as an inhibitor of apoplastic maize cysteine proteases. This activity, which is essential for *U. maydis* virulence, is mediated by a conserved motif of Pit2 that represents a novel protease inhibitor domain.

## Results

### Pit2 does interact with maize cysteine proteases

**Figure 1:**
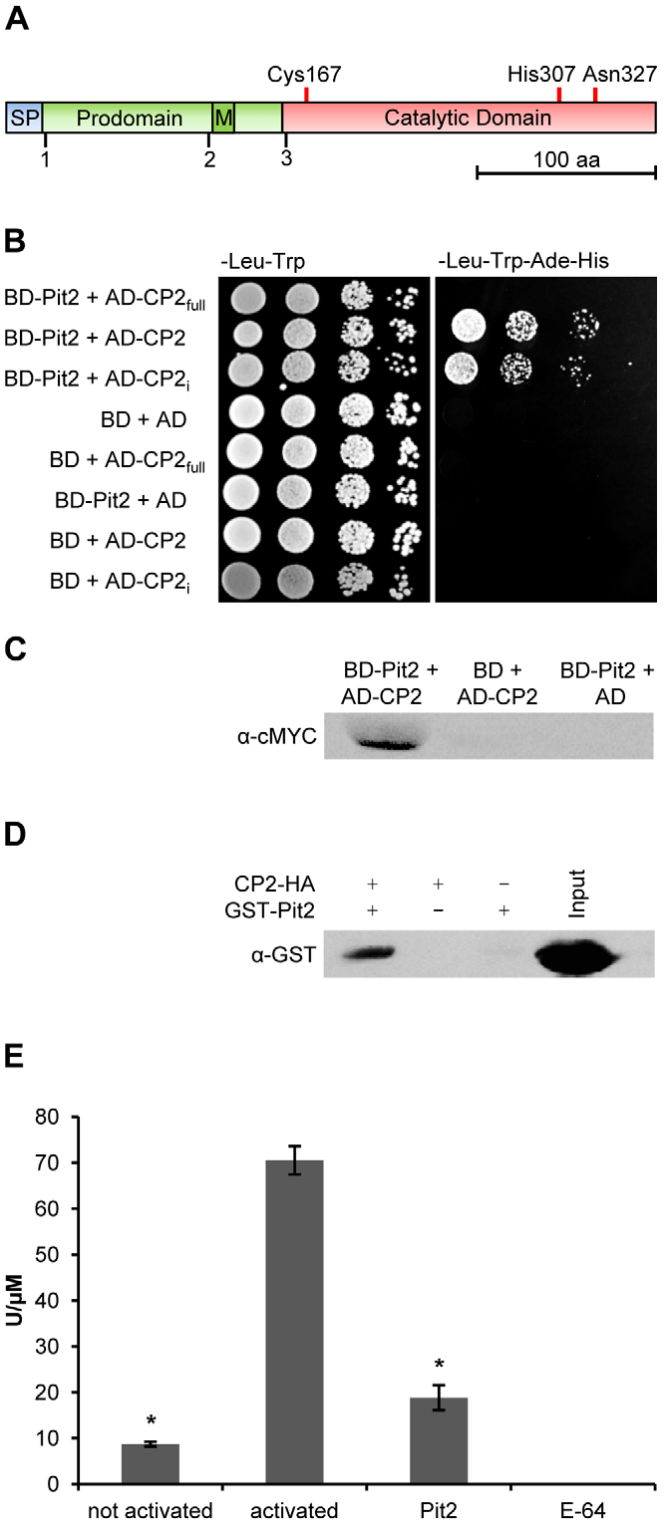
Interaction of Pit2 and the maize protease CP2. (**A**) Domain structure of the maize cysteine protease CP2, which was identified as Pit2 interaction partner in a yeast-two-hybrid screen. SP: signal peptide, M: minichain, typical for aleurain-like proteases [29]. Cys167, His307, Asn327 form the catalytic triad. Numbers (1–3) indicate sequence start of CP2 constructs (1) used for heterologous CP2 expression in *E. coli*, (2) used for Y2H experiments, (3) the CP2 fragment identified in the initial Y2H library screen. (**B**) Y2H interaction tests of Pit2 with different versions of CP2. Pit2 does not interact with CP2_full_ (Full-length protease including N-terminal prodomain, corresponding to (1) shown in (A)) but interacts with inactivated CP2_i_ (Catalytic triad residues were replaced by Glycine). BD: Gal4 Binding Domain. AD: Gal4 Activation Domain. (**C**) Co-immunoprecipitation shows interaction of Pit2 and CP2 fusion-proteins isolated from yeast cells. (**D**) Co-immunoprecipitation of *E. coli* expressed GST-Pit2 and HA-tagged CP2 protein that was expressed in *N. benthamiana* (see Figure 3). (**E**) Fluorescence based protease assay shows activity of recombinant CP2 that was purified from *E. coli* and its inhibition by Pit2 as well as the specific small-molecule cysteine protease inhibitor E-64. not activated: purified CP2 shows only very low activity. activated: activity of CP2 after activation by pH-shift and treatment with 10 mM pepsin (see material and methods for further details). Pit2: Addition of 10 µM Pit2 resulted in significant reduction of CP2 activity. 2.5 µM E-64 inhibited CP2. The experiment was carried out in three independent replicates; error bars represent SEM; P values were calculated by an unpaired t test. *P<0.05.

To identify interaction partners of Pit2, we performed a yeast-two-hybrid (Y2H) screen using a cDNA library of *U. maydis* infected maize leaves [28]. The coding region of *pit2* without its N-terminal secretion signal was cloned into the bait vector and co-transformed with the cDNA library into yeast cells. For 100 clones that were isolated from high-stringency selection medium, the interacting sequences were analyzed by restriction digest and subsequent sequencing. Strikingly, all Pit2-interacting clones corresponded to the maize protease CP2 (corn cysteine protease 2) (Figure 1A; Figure S1). CP2 is an aleurain like cysteine protease similar to CYP3 of tomato and AALP of *A. thaliana* [25], [29]. The protease contains a secretion signal, an N-terminal prodomain domain and a conserved protease domain. To confirm the Pit2-CP2 interaction, the coding region of CP2 without its secretion signal was cloned into a prey vector and tested for interaction with Pit2. However, expression of full-length CP2 did not recover growth on high-stringency medium (Figure 1B). By contrast, the interaction was restored when CP2 was expressed without its inactivating N-terminal prodomain (Figure 1B), confirming the result from the Y2H screening approach. To further analyze the Pit2-CP2 interaction, point mutations were introduced into the active site of CP2. To this end, residues C167, H307 and N327, which form the catalaytic triade of CP2 (Figure 1A) were exchanged to glycine. The resulting mutant protein CP2_i_ was co-expressed with Pit2 in yeast to test for interaction. Growth of the respective strain was observed on selective medium, indicating that activity of the protease is not required for the Pit2-CP2 interaction (Figure 1B). To verify the Pit2-CP2 interaction, co-immunoprecipitation with total protein extract of yeast cells expressing both proteins was performed (Figure 1C). Using anti-HA matrix, Pit2-MYC was co-immunoprecipitated by the HA-tagged CP2 but not by HA-tagged activation domain, which was used as negative control (Figure 1C). In addition, a co-immunoprecipitation experiment was done using *E. coli* expressed GST-Pit2 and CP2-HA that was expressed in *Nicotiana benthamiana* (see below). Also this experiment confirmed the direct interaction of Pit2 and the maize cysteine protease CP2 (Figure 1D).

### Pit2 functions as an inhibitor of maize cysteine proteases

In a complementary approach, recombinant Pit2 was expressed in *E. coli* with an N-terminal GST-tag that was removed by PreScission protease cleavage and subsequent gel filtration (Figure S2). His-tagged CP2 was produced in *E. coli* and re-solubilized from purified inclusion bodies (Figure S3). The immature CP2 was then activated by a pH shift and addition of 10 nM pepsin. Protease-activity was determined using the fluorescent substrate Z-Phe-Arg-AMC. Recombinant CP2 showed an activity of about 70 U/µM, which was inhibited by addition of the specific small molecule cysteine protease inhibitor E-64 [30] (Figure 1E). Moreover, addition of 10 µM Pit2 resulted in an 85% inhibition of CP2 (Figure 1E). These results suggest that Pit2 inhibits the maize cysteine protease CP2 by direct interaction.

**Figure 2:**
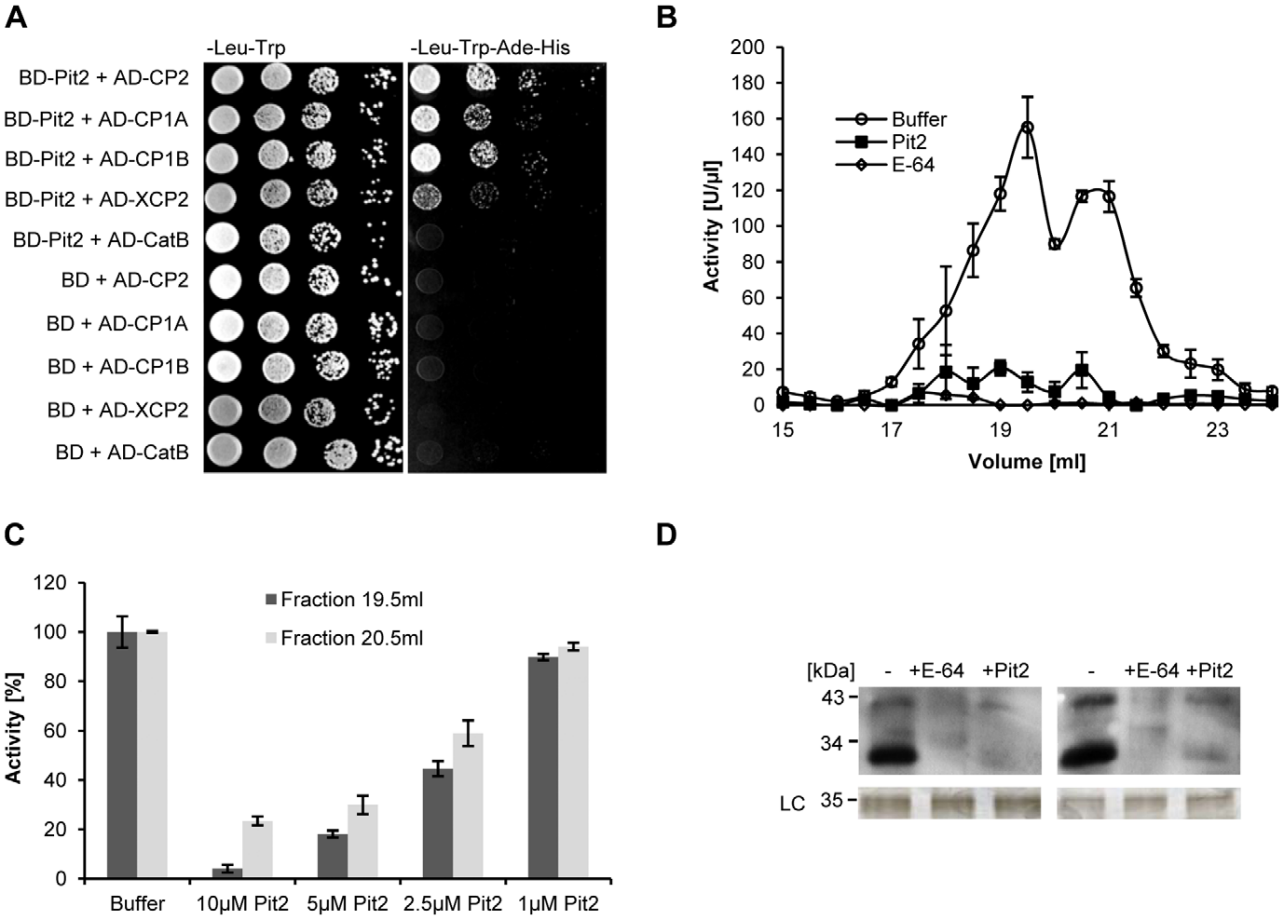
Pit2 interacts with different apoplastic maize cysteine proteases and inhibits their activity. (**A**) Y2H assay showing interaction of Pit2 with the maize cysteine proteases CP1A, CP1B, CP2 and XCP2 but not with CatB. BD: Gal4 Binding Domain. AD: Gal4 Activation Domain. (**B**) Protease activity in fractionated apoplastic fluid of maize leaves after treatment with SA. Highest activity was observed in fractions at elution volume 19.5 ml and 20.5 ml. Protease activity was inhibited by treatment with 10 µM recombinant Pit2 or 5 µM E-64, respectively. (**C**) Protease activity in apoplastic fluid fractions 19.5 ml (dark grey) and 20.5 ml (light grey). Application of 1–10 µM recombinant Pit2 resulted in a concentration-dependent inhibition of protease activity. **D**) Activity based protein profiling of cysteine proteases in apoplastic fractions 19.5 ml and 20.5 ml using the specific probe DCG-04 shows inhibition of the apoplastic proteases by Pit2 and E-64. LC: loading control; error bars represent SEM.

CP2 is amongst the five cysteine proteases, which we recently identified in the apoplast of maize leaves as crucial components of the SA-associated defense [25]. Besides CP2, the apoplast of SA-treated maize leaves contained the proteases CP1A, CP1B, XCP2 and CathepsinBIII (CatB) [25]. To test whether Pit2 does also interact with these apoplastic cysteine proteases, their coding regions were cloned and tested individually for Pit2-interaction in Y2H assays. Pit2 showed strong interaction with CP1A and CP1B, two isoforms of the maize cysteine protease CP1 that share 95% identity in the protease domain. In addition, co-expression of Pit2 and XCP2 resulted in weaker growth on high stringency medium (Figure 2A). For CatB, no interaction with Pit2 in yeast was observed (Figure 2A).

Interaction of Pit2 with the apoplastic cysteine proteases suggests that Pit2 might act as an inhibitor of these enzymes. However, the observed interaction in the heterologous yeast system does not necessarily imply a biological activity of Pit2 on these proteases. We therefore tested the ability of Pit2 to inhibit the cysteine proteases in apoplastic fluid extracts of maize leaves. To this end, protease activity was directly tested in ion-exchange chromatography fractionated apoplastic fluids, using the fluorescent substrate Z-Phe-Arg-AMC. In this assay, Pit2 significantly inhibited the cysteine protease activity in all active fractions with an efficiency of 64.83% (18 ml) to 100% (21,5 ml) (Figure 2B). The two fractions with the highest protease activity (fractions 19.5 and 20.5, see Figure 2B) were treated with different concentrations of Pit2, which showed that the protease-inhibition appears in a concentration-dependent manner (Fig. 2C). In addition, activity based protease profiling using the cysteine-protease specific probe DCG-04 [31] confirmed the protease inhibition in these fractions, i.e. an addition of recombinant Pit2 blocked protease labeling by the probe (Fig. 2D). As a control, proteases were inhibited by E-64 (Fig. 2D). Silver staining of SDS gels showed full-length Pit2 protein and no degradation bands before as well as after co-incubation with the proteases, demonstrating that the effector does not serve as a protease-substrate (Figure S4)

**Figure 3:**
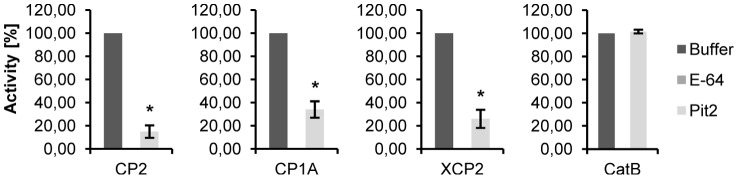
Activity of *N. benthamiana* expressed maize cysteine proteases and their inhibition by Pit2. Activity of CP2, CP1A, XCP2 and CatB that were transiently expressed in *N. benthamiana* using *A. tumefaciens* mediated transformation. Protease activity of CP2, CP1A and XCP2 was significantly inhibited by 10 µM of recombinant Pit2, while activity of CatB was not sensitive to Pit2. E-64 (5 µM) inhibited all four maize proteases. The experiment was carried out in three independent replicates; error bars represent SEM; P values were calculated by an unpaired t test. *P<0.05.

To further study the specificity of Pit2, one of the CP1 isoforms (CP1A), CP2, XCP2 and CatB were heterologously expressed in *Nicotiana benthamiana* using *Agrobacterium tumefaciens* mediated transformation (for details see methods section). Activity of the maize proteases was determined by fluorescence protease assays. The activity of all enzymes was entirely inhibited by the addition of 5 µM E-64, demonstrating the specificity of the protease activity (Figure 3). Similarly, recombinant Pit2 significantly inhibited the activity of CP1A, CP2 and XCP2 (Figure 3). By contrast, activity of CatB was not inhibited by Pit2, which correlates with the observation in the Y2H assay, where these two proteins did not show interaction (Figure 2A). Together, we conclude from these data that Pit2 functions as an inhibitor of the apoplastic maize cysteine proteases CP1A, CP2 and XCP2 but not CatB.

### A conserved motif is required for function of Pit2

**Figure 4:**
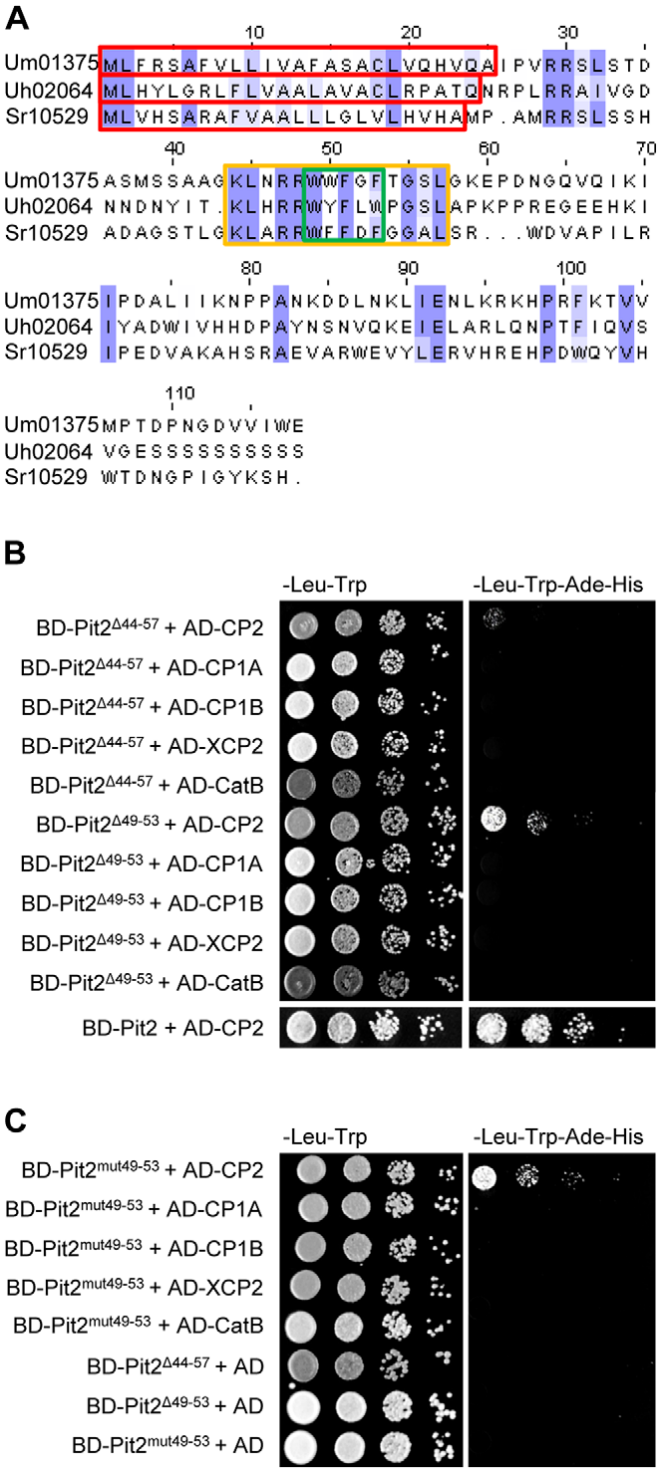
Sequence conservation of Pit2 in related smut fungi and the role of a conserved domain for interaction with cysteine proteases. (**A**) Alignment of *U. maydis* Pit2 (Um01375) and orthologs from the barley covered smut fungus *Ustilago hordei* (Uh02064) and the maize anther smut *Sporisorium reilianum* (Sr10529). Red box marks predicted secretion signals. Magenta labels identical (dark) and similar (light) amino acid residues. Orange box shows a 14 amino acid, highly conserved sequence stretch (residues 44–57). Green box marks the four aromatic residues that were addressed by targeted mutagenesis (see Table 1). (**B**) Y2H analysis of Pit2 mutants carrying deletions of the conserved domain shown in (A). (**C**) Y2H of Pit2 mutants carrying mutations of the conserved domain shown in (A) and Table 1. BD: Gal4 Binding Domain. AD: Gal4 Activation Domain.

The genomes of two smut fungi related to *U. maydis*, the maize anther smut *Sporisorium reilianum* and the barley covered smut *Ustilago hordei*, have recently been sequenced [32], [33]. Both genomes contain each one coding sequence with a significant similarity to the *U. maydis pit2* gene. These *pit2* orthologs, however, show only weak conservation with the encoded proteins sharing only 33.8% (*S. reilianum*) and 27.5% (*U. hordei*) sequence identity to *U. maydis* Pit2, respectively. However, a sequence stretch of 14 amino acids that contains four aromatic residues is well conserved among the three sequences (Figure 4A). Based on this similarity pattern, we hypothesized that this conserved region may be involved in the function of Pit2, i.e. its ability to interfere with cysteine proteases. To test this assumption, different mutations were introduced into the *U. maydis pit2* gene to generate mutated versions of Pit2. In *pit2^Δ44–57^*, the whole conserved region was deleted, while *pit2^Δ49–53^* carries a deletion of the five central residues of the region. In *pit2^mut49–53^*, point mutations were introduced to exchange four aromatic residues in the motif to glycine and alanine, respectively (Table 1). The resulting Pit2 mutant versions were tested in Y2H assays for interaction with the maize cysteine proteases. Strikingly, both mutants that carried deletions within the conserved motif did not show any interaction with CP1A, CP1B, XCP2 and CatB (Figure 4B). Similarly, Pit2^mut49–53^ did not show any interaction with these proteases (Figure 4c). For CP2, however, both Pit2^mut49–53^ and Pit2^Δ49–53^ showed almost wild-type growth while for Pit2^Δ44–57^ only marginal growth was observed (Figure 4B,C).

**Table 1:** The conserved Pit2 motif and different mutations tested in this study.

Name	Sequence (Pit2 aa 44–57)
Pit2	**AAGKLNRRWWFGFTGSLGKE**
Pit2^Δ44–57^	**AAG--------------GKE**
Pit2^Δ49–53^	**AAGKLNRR-----TGSLGKE**
Pit2^mut49–53^	**AAGKLNRRGGAGGTGSLGKE**

**Figure 5:**
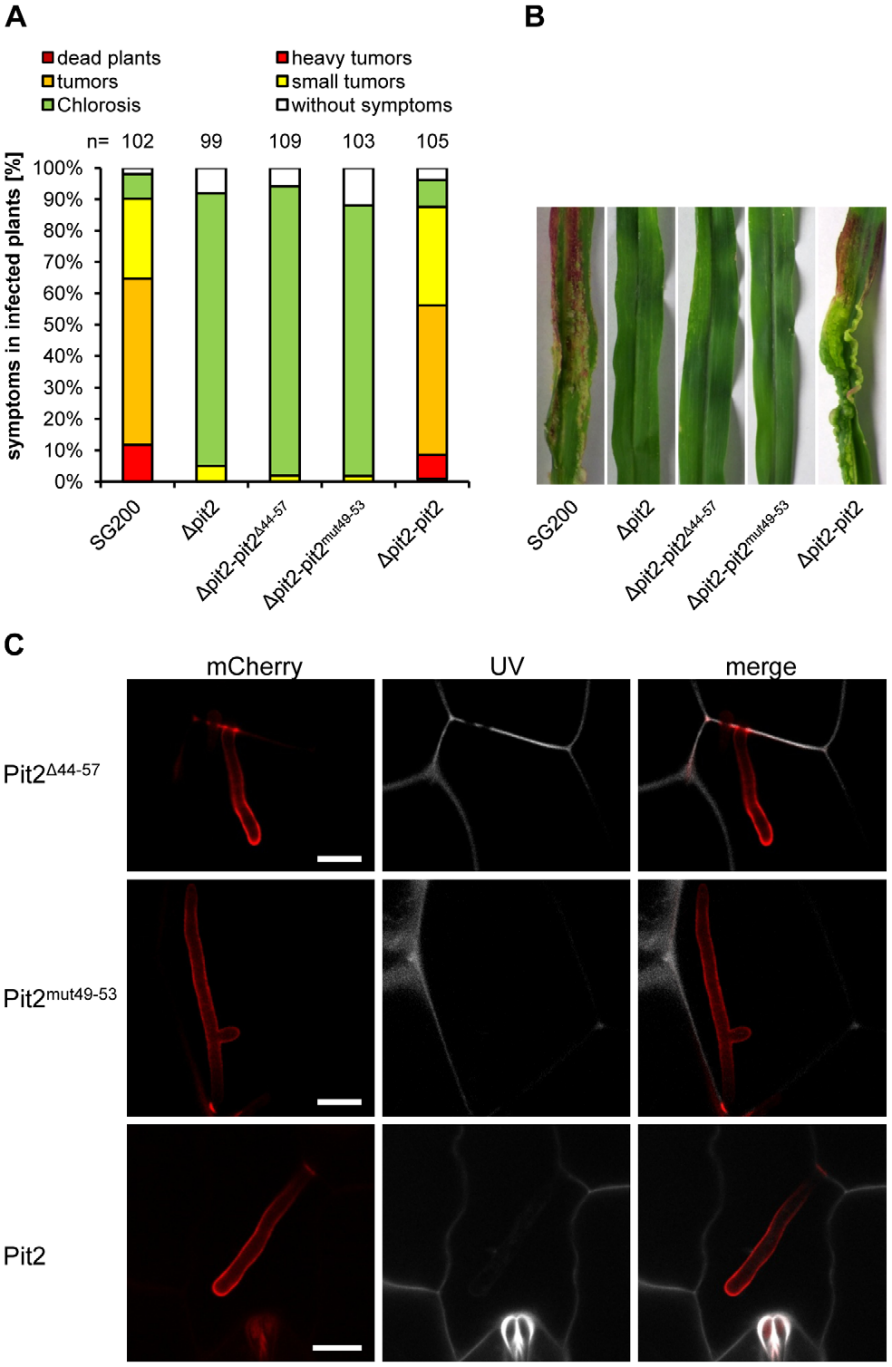
The conserved domain is required for function of Pit2 and virulence of *U. maydis*. (**A**) Disease rating of maize seedlings 12 days after infection with *U. maydis*. SG200: virulent *U. maydis* strain showing wild type disease symptoms. Δpit2: strain SG200Δpit2, a *pit2* deletion mutant derived from strain SG200 [27]. Δpit2_pit2^mut49–53^: Strain SG200Δpit2 complemented with pit2^mut49–53^ (see Figure 4A, Table 1). Δpit2_pit2^Δ49–53^: Strain SG200Δpit2 complemented with pit2^Δ49–53^ (see Figure 4A, Table 1). Δpit2_pit2: Δpit2 mutant complemented with wild-type *pit2* [27]. (**B**) Disease symptoms on maize leaves 12 days after infection with the *U. maydis* strains shown in (A). (**C**) Confocal images of intracellular *U. maydis* hyphae showing secretion of mCherry-tagged wild type Pit2 (lower panel) as well as the mutated Pit2 versions Pit2^Δ44–57^ (upper panel) and Pit2^mut49–53^ (middle panel). Red: mCherry fluorescence. Grey: UV-laser induced autofluorescence of maize cell walls. Bars: 10 µm.

To test whether this results correlate with the biological activity of Pit2, *pit2^Δ44–57^*as well as *pit2^mut49–53^* were expressed in the *U. maydis Δpit2* mutant under control of the native *pit2* promoter. The resulting *U. maydis* strains SG200Δpit2-pit2^Δ44–57^and SG200Δpit2-*pit2^mut49–53^* were infected to maize seedlings and scored for tumor formation. As a positive control, *U. maydis* strain SG200Δpit2-pit2 was used, which was complemented with the wild type *pit2* gene [27]. As expected, expression of wild type *pit2* fully restored tumor formation of the *pit2* deletion mutant (Figure 5A). By contrast, both SG200Δpit2-pit2^Δ44–57^ as well as SG200Δpit2-pit2^mut49–53^ were unable to induce the formation of plant tumors and were not distinguishable from the *pit2* deletion mutant (Figure 5A,B). To allow the localization of the proteins *in-planta*, *U. maydis* strains were produced that expressed mCherry-tagged Pit2 fusion-proteins. Life cell imaging using confocal microscopy revealed that expression and localization of the mutated Pit2 fusion-proteins was indistinguishable from that of mCherry-tagged wild type Pit2 (Figure 5C). Both the mCherry-tagged Pit2^Δ44–57^ and Pit2^mut49–53^ proteins were found to accumulate in the biotrophic interface, surrounding the intercellular hyphae. The fluorescent signals were spreading in the intercellular spaces around sites of cell-to-cell penetrations (Figure 5C), which confirms our previous localization of wild type Pit2 [27]. In addition, *U. maydis* strains expressing mCherry-HA tagged fusion proteins of wild type Pit2, Pit2^Δ44–57^ and Pit2^mut49–53^ were infected to maize leaves for subsequent α-HA western detection of the respective fusion-proteins. All three Pit2-variants were detected at the expected size as full-length proteins (Figure S5), demonstrating the stability of the fusion-proteins being secreted by *U. maydis* during plant colonization. From these results we conclude that the conserved motif is not required for *in-planta* expression and localization of Pit2, but is essential for the virulence function of this effector.

### Protease inhibition by Pit2 is mediated by the conserved inhibitor domain

**Figure 6:**
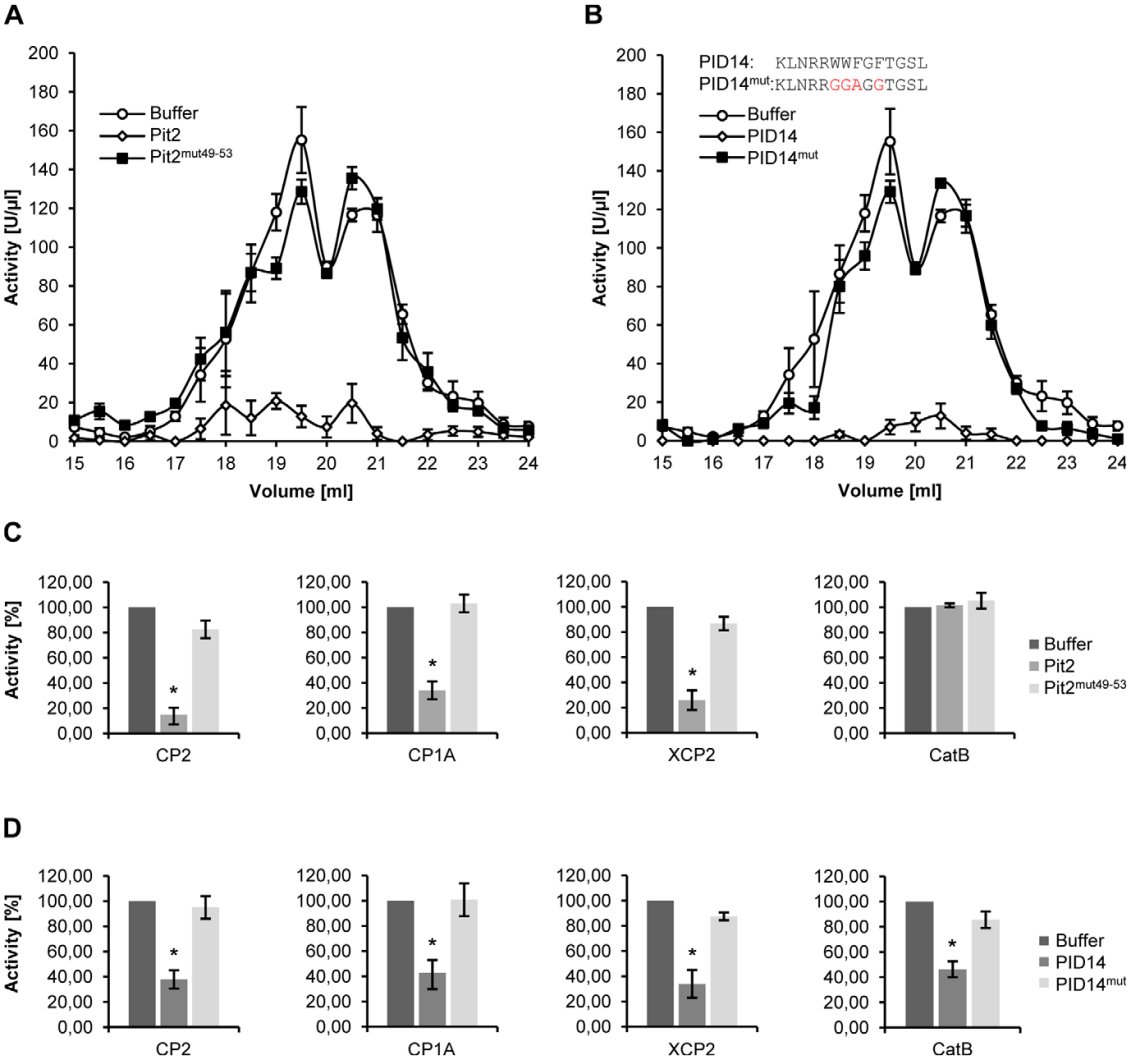
Specific inhibition of apoplastic maize proteases by Pit2 and the protease inhibitor domain PID14. (**A**) Protease activity in fractionated apoplastic fluid of maize leaves is inhibited by 10 µM Pit2 but not by the same concentration of Pit2^mut49–53^. (**B**) Protease activity of the apoplastic fluid fractions shown in (A) is efficiently inhibited by 10 µM of the PID14 peptide, while the same concentration of peptide PID14^mut^ did not significantly influence protease activity. (**C**) Activity of *N. benthamiana* expressed maize proteases CP2, CP1A and XCP2 is inhibited by 10 µM recombinant Pit2 (as shown in Figure 3) but not by the same concentration of recombinant Pit2^mut49–53^. (**D**) Activity of *N. benthamiana* expressed maize proteases CP2, CP1A, XCP2 and CatB is inhibited by 10 µM of the PID14 peptide but not by the same concentration of recombinant PID14^mut^. The experiment was carried out in three independent replicates; error bars represent SEM; P values were calculated by an unpaired t test. *P<0.05.

The conserved domain in Pit2 is required for its virulence function as well as for the interaction with cysteine proteases in Y2H assays. Next, we wanted to test whether these observations are linked to the actual ability of Pit2 to inhibit cysteine protease activity. To this end, Pit2^mut49–53^ protein was purified from *E. coli* under the same conditions that were used to produce native Pit2 protein. The recombinant Pit2^mut49–53^ protein was tested for its ability to inhibit apoplastic maize cysteine proteases. By contrast to the native protein, Pit2^mut49–53^ did not cause a significant inhibition of protease activity when being applied at similar concentrations (Figure 6A). From these results we conclude that the conserved 14 amino acid motif is the functional domain of Pit2, whose mutation causes a loss of function.

Given this crucial role of the conserved motif for Pit2 function as a protease inhibitor, we hypothesized that this motif might act as the functional domain of Pit2. We therefore tested synthetic peptides of the putative protease inhibitor domain in protease activity assays. One peptide (PID14, Protease Inhibitor Domain 14) contained the 14 residues of the conserved motif. A second peptide (PID14^mut^) corresponds to the mutated motif represented in Pit2^mut49–53^. Strikingly, PID14 inhibited the apoplastic cysteine-proteases with a similar efficiency as it was observed for Pit2 (Figure 6B). By contrast, PID14^mut^ did not cause a significant reduction of protease activity (Figure 6B). Finally, each Pit2^mut49–53^, PID14 and PID14^mut^ were tested for suppression of the *N. benthamiana* expressed proteases CP1A, CP2, XCP2 and CatB. In contrast to native Pit2, Pit2^mut49–53^ did not inhibit any of the recombinant proteases (Figure 6C). For PID14, significant inhibition of all proteases, including CatB, was observed, while PID14^mut^ did not show any effect on protease activity (Figure 6D).

**Figure 7:**
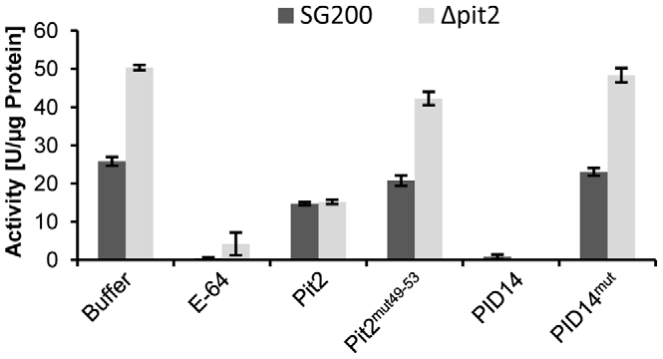
Protease activity in apoplastic fluids of maize plants infected with *U. maydis*. Apoplastic fluid was isolated from maize leaves two days after infection with *U. maydis* strain SG200 or SG200Δpit2, respectively. Buffer: Apoplastic fluid extracts showing increased protease activity in SG200Δpit2 infected maize plants compared to SG200 wild type infections. E-64: Activity after treatment with 5 µM E-64. Pit2: Treatment with 10 µM recombinant Pit2 reduced protease activity in both samples below wild-type infected level. The same concentration of Pit2^mut49–53^ did not cause reduction of protease activity. PID14: Treatment with 10 µM of the PID14 peptide completely inhibited protease activity; PID14^mut^ did not inhibit proteases. The experiment was carried out in three independent replicates; error bars represent SEM.

In an additional approach, the apoplastic fluids of maize leaves that had been infected either with *U. maydis* wild type (SG200) or the *Δpit2* mutant were isolated and tested for protease activity. Extracts of *Δpit2* infected tissue showed an about 2-fold increased protease activity when compared to that of *U. maydis* wild type infected material (Figure 7). This demonstrates that absence of Pit2 in the *U. maydis* - maize interaction causes a significant increase of cysteine protease activity in the infected tissue.

As expected, the low protease activity in wild type infected samples was only marginally reduced by application of recombinant Pit2, as this material already contained the native, *U. maydis-*secreted Pit2 (Figure 7). By contrast, the elevated protease activity in *Δpit2* infected samples was reduced by recombinant Pit2 to a residual activity level similar to that of wild type infected material. Treatment of these samples with Pit2^mut49–53^ again did not cause a significant effect. Similarly, PID14^mut^ did not cause a detectable inhibition of the protease activity, confirming the crucial role of the conserved inhibitor domain (Figure 7). Interestingly, PID14 almost completely inhibited maize protease activity, even below the level obtained by native Pit2. This observation is in line with the finding that PID14, in contrast to full length Pit2, also inhibits CatB, indicating that the target specificity found for Pit2 is not evident in case of PID14.

## Discussion

In this study we could demonstrate that the secreted *U. maydis* effector Pit2 acts as an inhibitor of apoplastic maize cysteine proteases to suppress host immunity. Inhibitory activity of Pit2 and therefore also *U. maydis* virulence depends on a 14 amino acid sequence motif that shows a high degree of conservation in related fungal pathogens. Moreover, this motif acts as a protease inhibitor domain that is sufficient to suppress maize cysteine proteases.

### Inhibition of apoplastic cysteine proteases by Pit2

In a Y2H screen with a cDNA library from *U. maydis* infected maize leaves, the cysteine protease CP2 was identified as interaction partner of Pit2. Given the previous finding of CP2 being one of the five apoplastic cysteine proteases that are crucial components in defense activation in maize, this result was a striking hint towards the function of Pit2 [25]. Although they were not identified in as interactors in the initial Y2H screen, the individual interaction test showed that Pit2 also binds to CP1A/B and XCP2, but not to the protease CatB. In addition, this result was also in line with the localization of Pit2 in the biotrophic interface and the apoplastic space at cell-to-cell penetrations [27]. At present, the exact mode of interaction, i.e. the binding site of Pit2 at the protease is unknown. However, the observation that presence of the complete N-terminal pro-domain of CP2 blocks the interaction suggests that the protease domain needs to be accessible for interaction. Together with the concentration-dependent inhibition of CP2 as well as apoplastic protease fractions, this argues for Pit2 being a competitive inhibitor of its target enzymes. On the other hand, enzymatic activity of the protease seems not to be required, since mutation of the catalytic triad of CP2 did not impair interaction. It will be subject of future approaches to determine the interaction on the molecular level by means of further mutational analyses and crystallization of the Pit2-CP2 protein complex.

Besides binding of CP2, Pit2 also does interact with the two isoforms of CP1 (CP1A, CP1B) as well as XCP2 but not with CatB. These interaction data correlate with the finding that Pit2 inhibits the activity of CP1, CP2 and XCP2 but not CatB. Similarly, in maize apoplastic fluids treatment with Pit2 typically resulted in some residual activity that could not be inhibited by the effector but was sensitive to E-64. This indicates a specificity of Pit2 in its interaction with cysteine proteases. Off note, this is also in line with the observation that commercial papain, which does also belong to the CP1 class of cysteine proteases, cannot be inhibited by recombinant Pit2 (data not shown). This specificity in protease targets is in contrast to the maize cystatin CC9, which does interact with all apoplastic maize cysteine proteases, completely inhibits apoplastic cysteine protease activity and also inhibits papain [25], [29]. It is tempting to speculate, why compatibility in the maize - *U. maydis* interaction depends on inhibition of apoplastic cysteine proteases both by the host side (by CC9) and by the pathogen side (by Pit2). In this regard it should be mentioned that all attempts to complement CC9 and Pit2 for each other were not successful (data not shown). This situation argues for the requirement of a temporal and spatial specificity by the individual inhibitors, i.e. CC9 is induced during the initial penetration phase and localizes in the apoplast, while Pit2 is required mainly during later stages and mainly accumulates in the biotrophic interface.

### A conserved motif functions as protease inhibitor domain

The Pit2 effector shows only weak conservation in related smut fungi and there are no significant orthologs in available sequences outside the group of *Ustilaginales*. Such a diversification of effectors is a well described phenomenon, resulting from the evolutionary pressure that originates from the need of proteins that interact with host targets to avoid recognition by the plant immune system [32], [34]. The mature Pit2 effectors from *U. maydis*, *S. reilanum* and *U. hordei* share only 15 identical residues. Of these, eight are located in the 14 aa conserved region that turned out to be the functional protease-inhibitor domain of Pit2. Evidence for the function of the PID14 is given by the mutational analyses that resulted in: i) loss of Pit2 – protease interaction in Y2H, ii) loss of protease inhibition of recombinant protein in the *in-vitro* assays and iii) loss of virulence, i.e. a *Δpit2* phenotype when the mutated versions were expressed in *U. maydis*. This line of evidence is further supported by the finding that the PID14 alone serves as a protease inhibitor and this activity is sensitive to mutation of the core aromatic residues within PID14. In contrast to full length Pit2, the PID14 completely blocks protease activity in apoplastic fluid of SG200 and *Δpit2* infected maize plants and also inhibits CatB, which is not sensitive to Pit2. This loss of target specificity could indicate that the flanking regions around the PID14 determine the target specificity of Pit2. Given the high diversity of these regions amongst UmPit2, UhPit2 and SrPit2 one could speculate whether the individual Pit2-orthologues have different target proteases, which may reflect the different host plants and infection styles of the three pathogens.

Protease inhibitors of the oomycete *P. infestans* show typical features that classify them into Kazal-like proteins for serine protease inhibitors or cystatin-like proteins for cysteine protease inhibitors, respectively [7], [10]. The *C. fulvum* Avr2 that inhibits the tomato cysteine proteases Rcr3 and Pip1 in a non-competitive manner is functionally different from phytocystatins, although it shares some sequence motifs [35]. The most remarkable structural features of Avr2 are the four disulphide bridges that are formed amongst the eight cysteine residues of the inhibitor [35]. This is in contrast to Pit2, which does not contain a cysteine residue in the mature protein and also the PID14 does not show conspicuous features of known inhibitor proteins. Therefore, Pit2 represents a novel type of protease inhibitor.

Given the crucial role of the PLCPs for apoplastic immunity in maize, it is surprising that there is no functional redundancy to Pit2 in *U. maydis*. This is reflected by the dramatic phenotype of the tested *pit2* mutants, where loss of the inhibitory function of Pit2 causes a complete loss of fungal virulence. The high variability of Pit2 orthologs in the related smut species *S. reilanum* and *U. hordei* indicates an evolutionary pressure on this effector, which underlines the importance of protease inhibition for these biotrophic pathogens. Recent findings identified the tomato cysteine protease RCR3 being targeted by effectors of at least three species: the *C. fulvum* effector Avr2, the secreted *P. infestans* cystatins EPIC1 and EPIC2B and the allergen-like effector Gr-VAP1 that is secreted by the nematode *Globodera rostochiensis* [9], [36]. These results illustrate the interactions of microbial inhibitors with apoplastic PLCPs as crucial determinant in biotrophic interactions. Therefore it will be important to study whether modulation of the apoplastic PLCPs in maize by the endogenous inhibitor CC9 [25] as well as by effectors like Pit2 is also crucial for other interactions, e.g. infection by hemibiotrophic or necrotrophic pathogens. Another challenge will be to understand, how the apoplastic PLCPs trigger defense gene activation. At present, the targets of these proteases and the downstream signaling components remain elusive. Therefore, the most intriguing questions are about the nature of the signals that are released upon PLCP activation in the apoplast, and how these signals are perceived by the plant.

The apoplastic space appears as a molecular battlefield where (in-) activation of defense-associated host enzymes decides on compatibility or host resistance. Future studies will aim to elucidate the molecular basis of Pit2-mediated protease inhibition. Solving the crystal structure of PID14/Pit2-protease complexes will aid to define the residues that determine the function of the inhibitor domain and illustrate the structural requirements for the cysteine protease inhibitor. This could enable the prediction of functionally conserved peptides in other plant pathogens as well as their target proteases, which are likely to be crucial determinants in apoplastic plant immune responses.

## Materials and Methods

### Strain and plasmid constructions

Standard molecular biology methods were used according to [37]. Oligonucleotides that were used for PCR are shown in Table S1. For expression of Pit2 in *E. coli*, um01375 was amplified by PCR without signal peptide using cDNA generated from SG200 infected maize plants as template. Next, the PCR-product was digested with *Xho*I and *EcoR*I and ligated in plasmid pRSET-GST-PP [38] to obtain pRSET-GST-PP-Pit2. To generate pRSET-GST-PP-Pit2^mut49–53^, site directed mutagenesis was performed according to the instructions of the QuikChange Multi Site-Directed Mutagenesis Kit (Stratagene, Santa Clara, USA) using Primer OPit2^mut49–53^ and pRSET-GST-PP-Pit2 as a template. To obtain pET22b-CP2, CP2 (NP_001105479.1) was amplified by PCR from maize cDNA excluding the coding region for the signal peptide and cloned into plasmid pET22b (Novagen/Merck, Darmstadt, Germany) via *Nde*I and *Hind*III. To construct pGBKT7-Pit2 and pGBKT7-Pit2^mut49–53^, Pit2 and Pit2^mut49–53^ were amplified from plasmids pRSET-GST-PP-Pit2 and pRSET-GST-PP-Pit2^mut49–53^, respectively. Next, the PCR-products were digested with *Nco*I and *EcoR*I and ligated in plasmid pGBKT7 (Clontech, Mountain View, USA). To obtain pGBKT7-Pit2^Δ44–57^ and pGBKT7-Pit2^Δ49–59^, inverse-PCR was performed using pGBKT7-Pit2 as a template and the PCR-product was then blunt-end-ligated. To obtain pGADT7-CP2_full_, CP2 was amplified from maize cDNA excluding the DNA-region coding for the signal-peptide, digested with *Nde*I and *Bam*HI and ligated in pGADT7 (Clontech, Mountain View, USA). CP2 without the region coding for the signal-peptide and the prodomain was amplified from pGADT7-CP2_full_, digested with *Nde*I and *Bam*HI and ligated in pGADT7. CP1A, CP1B, XCP2 and CathepsinBIII (CP1A: NP_001148706.1, CP1B: NP_001149658.1, XCP2: NP_001149806.1, CathepsinBIII: NP_001150152.1) were amplified from maize cDNA excluding the DNA-regions coding for the respective signal-peptides and prodomains, digested with *Nde*I and *Bam*HI and ligated in pGADT7. To generate pGADT7-CP2_i_, site directed mutagenesis was performed according to the instructions of the QuikChange Multi Site-Directed Mutagenesis Kit (Stratagene, Santa Clara, USA) using primers targeting nucleotides coding for the active site of CP2 using pGADT7-CP2 as a template. To obtain p123-Ppit2-pit2^mut49–53^ and p123-Ppit2-pit2^mut49–53^-mCherry, site directed mutagenesis was performed according to the instructions of the QuikChange Multi Site-Directed Mutagenesis Kit (Stratagene, Santa Clara, USA) using primer OPit2^mut49–53^ and templates p123-Ppit2-pit2 or p123-Ppit2-pit2mCherry [27], respectively. To generate p123-Ppit2-pit2^Δ44–57^ and p123-Ppit2-pit2^Δ44–57^-mCherry, inverse-PCR was performed using p123-Ppit2-pit2 or p123-Ppit2-pit2-mCherry [27] as template, respectively. Blunt-end ligation was done with the purified PCR-product. Plasmids pGreenII 0029-CP2, -CP1A, -XCP2 and -CatB were constructed by amplifying the sequences of the respective proteases from maize cDNA followed by digestion with *Xba*I and *Sac*I and ligation in pGreenII 0029.

### Expression of Pit2/Pit2^mut49–53^ in *E. coli*

Plasmids pRSET-GST-PP-Pit2 and pRSET-GST-PP-Pit2^mut49–53^ were transformed into Tuner(DE3)pLysS Competent Cells (Novagen/Merck, Darmstadt, Germany). An overnight culture of the respective strains in dYT medium supplemented with 100 µg/ml Ampicillin and 34 µg/ml Chloramphenicol was diluted 1∶100 in eight batches à 200 ml dYT supplemented with 100 µg/ml Ampicillin and 34 µg/ml Chloramphenicol in 1 l Erlenmeyer flasks. Cells were incubated at 37°C and 200 rpm to an OD_600_ of 0.6, and then protein expression was induced by 1 mM IPTG. After 16 h at 16°C and 200 rpm, cells were harvested by centrifugation for 30 min at 6000 rpm and 4°C in a Sorvall SLA-3000 centrifuge. For cell lysis, the pellet was subjected to a freeze-thaw cycle and resuspended in 10 ml lysis buffer (140 mM NaCl, 10 mM Na_2_HPO_4_, 1.8 mM KH_2_PO_4_, 2.7 mM KCl, 0.5 mM EDTA, 1% Triton X-100, pH 7.4). After incubation for 20 min at room temperature the suspension was sonicated 5 times for 45 s, and insoluble cell debris were removed by centrifugation for 30 min at 20000 rpm and 4°C (Sorvall SS-34).

20 ml supernatant was applied on a flow through column (Thermo Scientific, Rockford, USA) loaded with 1 ml GST-sepharose (GE-Healthcare, Uppsala, Sweden) that was equilibrated with 3×10 ml PBS (140 mM NaCl, 10 mM Na_2_HPO_4_, 1.8 mM KH_2_PO_4_, 2.7 mM KCl, pH 7.3). The flow through was collected and applied to the GST-matrix two times followed by three washing steps with 10 ml PBS and one washing step with PreScission cleavage buffer (50 mM Tris-HCl pH = 7.5, 150 mM NaCl, 1 mM EDTA, 1 mM DTT). PreScission protease cleavage was performed by addition of 2 ml cleavage solution (4% protease stock (2000 U/ml) in PreScission cleavage buffer) and incubation for 16 h at 4°C. The supernatant was collected, the matrix was rinsed with 2 ml cleavage buffer and the supernatant was again collected and pooled together with the first one. The resulting protein solutions from four flow-through columns were pooled and concentrated via Amicon Ultra-4 devices (Millipore/Merck, Darmstadt, Germany) with a molecular weight cut-off of 3 kDa to a final volume of about 6 ml. The concentrated protein solution was then sterile filtrated, applied on a gel filtration column (HiLoad Superdex 200, GE-Healthcare, Uppsala, Sweden) equilibrated with storage buffer (50 mM Tris-HCl, pH7, 150 mM NaCl) and eluted with storage buffer. Fractions corresponding to the peak fractions for Pit2/Pit2^mut49–53^ were pooled and again concentrated via Amicon Ultra-4 devices (Millipore) to a volume resulting in an appropriate protein concentration. For long term storage, 10% Glycerol was added and aliquots were stored at −80°C.

### Expression and refolding of CP2

For expression of CP2, plasmid pET22b-CP2 was transformed into BL21(DE3)pLysS competent cells (Novagen/Merck, Darmstadt, Germany). Expression and cell lysis was performed as described for Pit2 except for using a different lysis buffer (50 mM Tris-HCl, pH 8.0, 150 mM NaCl, 5 mM EDTA). After centrifugation for 30 min at 20000 rpm and 4°C (Sorvall SS-34), the soluble fraction was discarded and the pellet was washed once with 10 ml of a buffer containing 50 mM Tris-HCl, pH 8.0, 5 mM EDTA and 0.1% Triton X-100, once with 10 ml of a buffer containing 50 mM Tris-HCl, pH 8.0, 5 mM EDTA and 2 M Urea and once with 10 ml water intermitted by centrifugation for 10 min at 20000 rpm and 4°C (Sorvall SS-34) respectively. Next, the pellet was resuspended in 10 ml of denaturing binding-buffer (20 mM Tris-HCl, pH 7.9, 500 mM NaCl, 20 mM Imidazol, 6 M Guanidin-HCl) and incubated for 1–3 h at 4°C on a rotation wheel until the pellet was resolved followed by centrifugation for 20 min at 20000 rpm and 4°C (Sorvall SS-34). The supernatant was applied to a column containing 1 ml of Ni-sepharose (GE-Healthcare, Uppsala, Sweden) equilibrated with 20 ml of denaturing binding-buffer and incubated for 1 h at 4°C on a rotation wheel. The flowthrough was discarded and the beads were washed three times with 10 ml of denaturing binding-buffer followed by three washing steps with 10 ml of a buffer containing 20 mM Tris-HCl, pH 7.9, 500 mM NaCl, 60 mM Imidazol and 6 M Guanidin-HCl, respectively. Elution of the unfolded protein was performed two times by applying 5 ml of elution-buffer (20 mM Tris-HCl, pH 7.9, 500 mM NaCl, 1 M Imidazol, 6 M Guanidin-HCl) and incubation for 15 min at room temperature. DTT was added to a final concentration of 10 mM and the solution was incubated on ice for at least 30 min before the protein concentration was determined according to [39]. Concentration was adjusted to 100 µg/ml with elution-buffer, followed by dialysis over night at 4°C and slight stirring against a 100-fold volume of refolding-buffer (50 mM Tris-HCl, pH 7.0, 30% Glycerol, 2.5 mM GSH, 1 mM GSSG, 5 mM EDTA, 150 mM NaCl). The refolded protein was then concentrated to a total volume of 10 ml using Amicon Ultra-4 devices (Millipore/Merck, Darmstadt, Germany) with a molecular weight cut-off of 10 kDa. The protein solution was then dialyzed two times against a 100-fold volume of storage buffer (50 mM Tris-HCl, pH 7.0, 150 mM NaCl) for 1 h at 4°C, respectively and again concentrated to an appropriate protein concentration.

### Protease activity assays

Dialyzed CP2 was centrifuged at 17000×*g* for 5 min at 4°C to remove precipitated protein. The supernatant was transferred to a new reaction tube and its pH was adjusted to 4.5 with 500 mM Na-Acetate. Porcine pepsin was added resulting in an enzyme/CP2 molar ratio of 1∶100. After 1 h incubation at RT the pH was increased to 6.0 using 1 M sodium phosphate to stop the activation process. Protease activity was measured using the fluorimetric substrate Z-Phe-Arg-7-amido-4-methylcoumarin (Z-Phe-Arg-AMC) (Sigma-Aldrich, Taufkirchen, Germany), which leads to release of fluorescence at 460 nm when cleaved by protease activity [40]. To determine the protease activity of refolded CP2, defined amounts of protein were incubated either with buffer, E-64 or Pit2 for 10 min in a total volume of 90 µl. 10 µl of 10 µM substrate were added to a final reaction volume of 100 µl. Fluorescence was monitored using a fluorometer (Safire, Tecan, Crailsheim, Germany).

Preparation and fractionation of apoplastic fluid from SA-treated, SG200-infected or SG200Δpit2-infected maize seedlings was performed according to [25].

Protease activity of apoplastic fluid fractions from SA-treated maize leaves was determined using substrate Z-Phe-Arg-AMC (Sigma-Aldrich, Taufkirchen, Germany). 10 µl of the respective fractions were incubated for 10 min at RT in 10 mM sodium phosphate buffer, pH 6.0, 150 mM NaCl, 1 mM EDTA, and 0.5 mM DTT in a total volume of 90 µl. Then 10 µl of 10 µM substrate was added and fluorescence was monitored using a fluorometer (Safire,Tecan, Crailsheim, Germany). Pit2, Pit2^mut49–53^, PID14, PID14^mut^, E-64 or storage buffer were preincubated with the apoplastic fluid fractions for 10 min.

For the activity based protein profiling of cysteine proteases in apoplastic fluid of maize seedlings, the specific probe DCG-04 was used [31]. 5 µl of respective apoplastic fluid fractions were incubated with 5 µM E-64 or 10 µM Pit2 for 30 min at room temperature. Then, 50 mM TrisHCl, pH7.0, 0.2 mM DTT and 2 µM DCG-04 were added followed by incubation at room temperature for 4 h. Containing proteins were precipitated by addition of 1 ml 100% Acetone, incubated at −20°C overnight and resolved in 100 µl 2× Laemmli loading buffer [41] prior detection via immunoblotting and -detection using strep-HRP (Sigma-Aldrich, Taufkirchen, Germany) as previously described by [31].

### Yeast transformation and two hybrid interaction assay

For yeast assays, strain AH109 (Clontech, Mountain View, USA) was used. Yeast transformation was done as described in the DUALmembrane starter kit manual (Dualsystems Biotech AG, Schlieren, Switzerland). The yeast two hybrid screen was performed following the instructions of the matchmaker yeast two hybrid manual (Clontech, Mountain View, USA) using 1 mg of bait-DNA (pGBKT7-Pit2) and 0.5 mg of library-DNA. All resulting yeast clones were tested by immunoblotting and -detection for expression of the respective proteins.

To perform a yeast dilution assay, 3 ml of selective medium (SD-Leu-Trp) was inoculated with a single colony of the respective yeast strain and incubated overnight at 28°C. OD_600_ was adjusted to 0.2 and the cells were grown to an OD_600_ of 0.6–0.8. Next, 1 ml of yeast culture was centrifuged for 10 min at 3500×*g* and the pellet was washed twice with 1 ml sterile water and finally resuspended in 1 ml sterile water. OD_600_ was adjusted to 1.0 with sterile water and 5 µl of this suspension, as well as 1∶10, 1∶100 and 1∶1000 dilutions, were applied on SD-Leu-Trp-plates (low stringency) as a growth control and on SD-Leu-Trp-Ade-His-plates (high stringency) to test for protein-protein-interaction.

### Immunoblotting and immunodetection

For immunoblotting, appropriate amounts of proteins were separated by SDS-PAGE [41] followed by transfer to a nitrocellulose membrane. After electroblotting, the membrane was saturated with 5% non-fat dry milk in TBS-T (50 mM Tris-HCl, 150 mM NaCl, pH 7.6, 0.1% Tween-20) for 1 h at room temperature. After blocking, the membrane was washed three times with TBS-T followed by incubation with the primary antibody (anti-HA antibody: 1∶10000, anti-c-*Myc* antibody: 1∶5000; Sigma-Aldrich, Taufkirchen, Germany) over night at 4°C. Membranes were washed three times prior to incubation for 1 h with HRP-conjugated secondary antibodies (anti-mouse, 1∶5000; Cell Signalling, Danvers, USA). Signals were detected by chemiluminscence detection using ECL Plus Western Blot detection reagent (GE-Healthcare, Uppsala, Sweden).

For co-immunoprecipitation of GST-Pit2, CP2-HA was heterologously expressed in *N. benthamiana* as described above. Infiltrated leaves were ground in liquid nitrogen and the resulting powder was mixed with Pit2 storage buffer. Leaf extract was then centrifuged at 10000 *g* and 4°C and subsequently sterile filtrated. GST-Pit2 expression was performed according to material and methods “Expression of Pit2/Pit2^mut49–53^ in *E. coli*” but GST-Pit2 and eluted from GST-Sepharose by adding 3 ml GST-Elution buffer (10 mM reduced glutathione in 50 mM Tris-HCL pH 8.0) instead of performing PreScission protease cleavage. 1 ml of leaf extract containing 1 mg/ml protein was incubated with 5 µM GST-Pit2 or Pit2 storage buffer, respectively, for 2 h at 4°C. Then 50 µl of Anti-HA Affinity Matrix (Roche Diagnostics Deutschland GmbH, Mannheim, Germany) was added and incubated over night on a rotation wheel. Next, the samples were centrifuged trough Pierce SpinColumns (Thermo Scientific, Rockford, USA), washed once with Pit2 storage buffer and protein was finally eluted by boiling with SDS loading buffer for 5 min. Detection was carried out by Western blot analysis using anti-GST-antibodies (1∶10000; Sigma-Aldrich, Taufkirchen, Germany) as described above.

For immunoprecipitation of Pit2-mCherry-HA, Pit2^mut49–53^-mCherry-HA and Pit2^Δ44–57^-mCherry-HA 40 plants were infected with the respective *U. maydis* strains, respectively. 3 dpi, the infected areas were cut out, directly frozen in liquid nitrogen, ground in liquid nitrogen and the resulting powder mixed with TBS buffer supplemented with Protease Inhibitor Cocktail Tablets (1 per 10 ml of Buffer; Roche Diagnostics Deutschland GmbH, Mannheim, Germany). After centrifugation for 15 min at 10000 *g*, the samples were filtrated and adjusted to a protein concentration of 0,5 mg/ml. Then 50 µl of Anti-HA Affinity Matrix (Roche Diagnostics Deutschland GmbH, Mannheim, Germany) was added and the samples were incubated on a rotation wheel at 4°C over night. Elution was performed according to the HA-Kit protocol (Pierce). Next, the samples were centrifuged trough Pierce SpinColumns (Thermo Scientific, Rockford, USA) and protein was finally eluted by boiling with SDS loading buffer for 5 min. Detection was carried out by Western blot analysis using anti-HA-antibodies (1∶10000; Sigma-Aldrich, Taufkirchen, Germany) as described above.

### Heterologous expression of maize proteases in *N. benthamiana*

Preparation and transformation of competent *Agrobacterium tumefaciens* cells was performed according to [42]. For all experiments, *A. tumefaciens* strain GV3101 was used.

Three days post *A. tumefaciens* infiltration, *N. benthamiana* leaves were harvested and lengthwise dissected by cutting out the petioles. The fresh weight was determined and the leaves were set to a vacuum chamber with sterile H_2_O and vacuum-infiltrated 3–5 times for 15 min at 200 mbar. Subsequently, the evacuated leaves were stacked and coiled up to fit in 20 ml-syringes that were put into 50 ml Greiner-tubes and centrifuged for 20 min at 2000×*g* and 4°C. Apoplastic fluid was aliquoted and stored at −20°C.

For protease activity assays, the ratio between obtained apoplastic fluid and fresh weight was calculated. Fluids of each sample were diluted to a ratio of 150 µl/g fresh weight with sterile water. Protease activity was determined using substrate Z-Phe-Arg-AMC (Sigma-Aldrich, Taufkirchen, Germany). 5 µl of the adjusted fluid were incubated for 10 min at room temperature (in buffer 54 mM sodium phosphate, pH 6.0, 600 mM NaCl, 4 mM EDTA, 2 mM DTT) in a total volume of 40 µl. Afterwards, 10 µl of 10 µM substrate was added and fluorescence was monitored using a fluorometer (Infinite M 200 Pro, Tecan, Crailsheim, Germany). The influence of Pit2, Pit2^mut49–53^, PID14, PID14^mut^ or E-64 was determined by pre-incubating the apoplastic fluid with the respective inhibitor prior substrate addition.

### Fungal strains, plant infections and confocal microscopy

All *U. maydis* strains used in this study (Tab. S2) were grown in YEPSL at 28°C (0.4% yeast extract, 0.4% peptone and 2% sucrose) and used in plant infections as described earlier [43]. Severity of disease symptoms was rated 12 days post infection as described previously [20].

Transformation of *U. maydis* and isolation of genomic DNA was performed according to [44]. All generated constructs were checked by sequencing before transformation of *U. maydis*. Resulting transformants were tested for single integration events in the desired loci by Southern blot analysis. Life cell imaging of fungal hyphae in maize tissue was performed on a TCS-SP5 confocal microscope (Leica, Wetzlar, Germany) as described previously [27].

## Supporting Information

Figure S1 Sequence comparison of the Genbank entry of the *CP2* gene (NP001105479.1) of the maize variety B73 and the sequence that was found in the Pit2 interaction screen by Y2H. Yellow: predicted signal peptide, brown: predicted propeptide, dark green: predicted catalytic domain, light green: SNPs in the Y2H-identified sequence, which is derived from the maize variety Early Golden Bantam.
(PDF)

Figure S2 Heterologous expression and purification of Pit2. Pit2 was expressed as a fusion protein C-terminal to GST. After cell lysis, the soluble fraction (S) was loaded on a column containing glutathion-sepharose. After several washing steps (W1–4), Pit2 was separated from GST by PreScission Protease cleavage using a specific PreScission protease cleavage site and hence eluted from the column (E). Further purification was performed by conducting gel filtration. Purity of the protein was afterwards tested by SDS-PAGE (FPLC).
(PDF)

Figure S3 Heterologous expression and purification of CP2. CP2 was expressed as a protein containing an N-terminal His-tag. After cell lysis, the soluble (S) and the pellet fraction (P) were separated. The pellet fraction was denatured using 6 M Guanidine-HCl and exposed to a Ni-sepharose column. After washing, the protein was eluted and refolded using an appropriate refolding buffer (see material and methods for further information). ref.: purity of the protein after refolding was tested by SDS-PAGE.
(PDF)

Figure S4 Silver stained SDS-PAGE showing FPLC purified Pit2 and Pit2^mut49–53^. Treatment of both Pit2 versions with apoplastic protease fraction 19.5 ml (Fraction 19.5; see Figure 2B) does not generate detectable degradation products.
(PDF)

Figure S5 Western Blot analysis to test stability of mCherry-HA tagged Pit2-fusion-proteins expressed by *U. maydis* during plant infection. Protein extracts of *U. maydis* infected maize leaves were probed using anti-HA-antibodies. Expected sizes for Pit2-mCherry-HA: 38.8 kDa, Pit2^Δ44–57^-mCherry-HA: 37 kDa, Pit2^mut49–53^-mCherry-HA: 38.4 kDa. No signal was detected in samples from maize leaves that were infected with the *U. maydis* wild type strain SG200.
(PDF)

Table S1 Oligonucleotides used in this study.
(DOCX)

Table S2 *U. maydis* strains used in this study.
(DOCX)
