# Supplementary material for: Compatibility in the Ustilago maydis–Maize Interaction Requires Inhibition of Host Cysteine Proteases by the Fungal Effector Pit2
Source: PLoS Pathog. 2013 Feb 14;9(2):e1003177. doi: 10.1371/journal.ppat.1003177 (PMC3573112; doi:10.1371/journal.ppat.1003177)
Supplement: Figure S1 — Sequence comparison of the Genbank entry of the CP2 gene (NP001105479.1) of the maize variety B73 and the sequence that was found in the Pit2 interaction screen by Y2H. Yellow: predicted signal peptide, brown: predicted propeptide, dark green: predicted catalytic domain, light green: SNPs in the Y2H-identified sequence, which is derived from the maize variety Early Golden Bantam. (PDF) [file ppat.1003177.s001.pdf]

# Figure S1

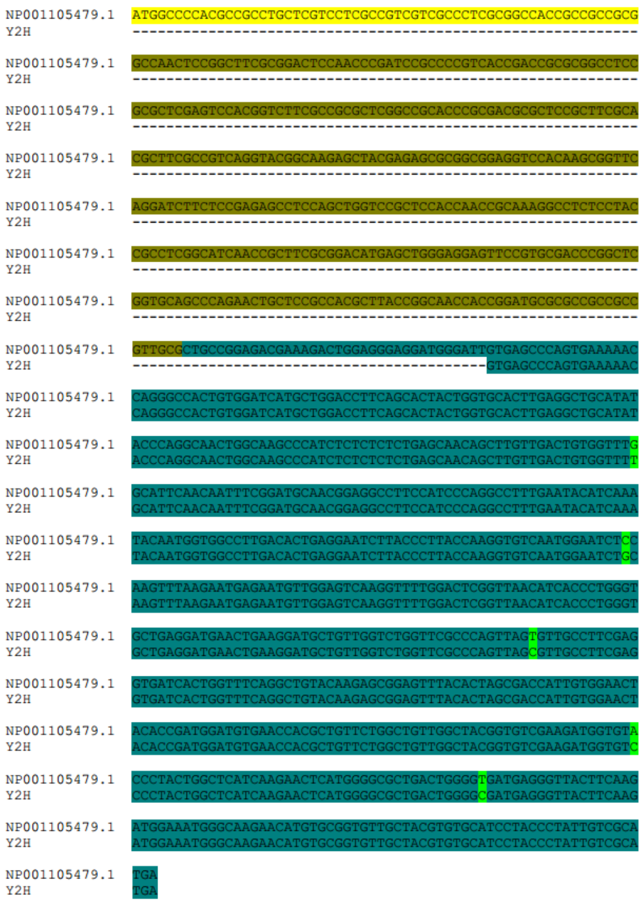

**Figure S1:** Sequence comparison of the Genbank entry of the *CP2* gene (NP001105479.1) of the maize variety B73 and the sequence that was found in the Pit2 interaction screen by Y2H. Yellow: predicted signal peptide, brown: predicted propeptide, dark green: predicted catalytic domain, light green: SNPs in the Y2H-identified sequence, which is derived from the maize variety Early Golden Bantam.
