# Supplementary material for: Compatibility in the Ustilago maydis–Maize Interaction Requires Inhibition of Host Cysteine Proteases by the Fungal Effector Pit2
Source: PLoS Pathog. 2013 Feb 14;9(2):e1003177. doi: 10.1371/journal.ppat.1003177 (PMC3573112; doi:10.1371/journal.ppat.1003177)
Supplement: Figure S2 — Heterologous expression and purification of Pit2. Pit2 was expressed as a fusion protein C-terminal to GST. After cell lysis, the soluble fraction (S) was loaded on a column containing glutathion-sepharose. After several washing steps (W1–4), Pit2 was separated from GST by PreScission Protease cleavage using a specific PreScission protease cleavage site and hence eluted from the column (E). Further purification was performed by conducting gel filtration. Purity of the protein was afterwards tested by SDS-PAGE (FPLC). (PDF) [file ppat.1003177.s002.pdf]

Figure S2

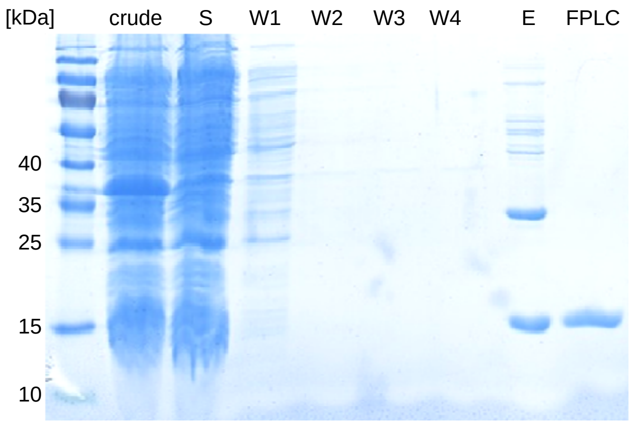

Figure S2: Heterologous expression and purification of Pit2. Pit2 was expressed as a fusion protein C-terminal to GST. After cell lysis, the soluble fraction (S) was loaded on a column containing glutathion-sepharose. After several washing steps (W1-4), Pit2 was separated from GST by PreScission Protease cleavage using a specific PreScission protease cleavage site and hence eluted from the column (E). Further purification was performed by conducting gel filtration. Purity of the protein was afterwards tested by SDS-PAGE (FPLC).
