# Supplementary material for: Compatibility in the Ustilago maydis–Maize Interaction Requires Inhibition of Host Cysteine Proteases by the Fungal Effector Pit2
Source: PLoS Pathog. 2013 Feb 14;9(2):e1003177. doi: 10.1371/journal.ppat.1003177 (PMC3573112; doi:10.1371/journal.ppat.1003177)
Supplement: Figure S3 — Heterologous expression and purification of CP2. CP2 was expressed as a protein containing an N-terminal His-tag. After cell lysis, the soluble (S) and the pellet fraction (P) were separated. The pellet fraction was denatured using 6 M Guanidine-HCl and exposed to a Ni-sepharose column. After washing, the protein was eluted and refolded using an appropriate refolding buffer (see material and methods for further information). ref.: purity of the protein after refolding was tested by SDS-PAGE. (PDF) [file ppat.1003177.s003.pdf]

Figure S3

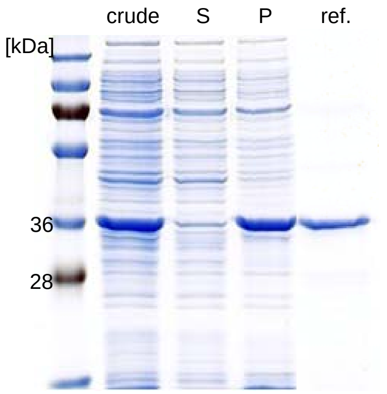

Figure S3: Heterologous expression and purification of CP2. CP2 was expressed as a protein containing an N-terminal His-tag. After cell lysis, the soluble (S) and the pellet fraction (P) were separated. The pellet fraction was denatured using 6M Guanidine-HCl and exposed to a Ni-sepharose column. After washing, the protein was eluted and refolded using an appropriate refolding buffer (see material and methods for further information). ref.: purity of the protein after refolding was tested by SDS-PAGE.
