# Supplementary material for: Compatibility in the Ustilago maydis–Maize Interaction Requires Inhibition of Host Cysteine Proteases by the Fungal Effector Pit2
Source: PLoS Pathog. 2013 Feb 14;9(2):e1003177. doi: 10.1371/journal.ppat.1003177 (PMC3573112; doi:10.1371/journal.ppat.1003177)
Supplement: Figure S4 — Silver stained SDS-PAGE showing FPLC purified Pit2 and Pit2mut49–53. Treatment of both Pit2 versions with apoplastic protease fraction 19.5 ml (Fraction 19.5; see Figure 2B) does not generate detectable degradation products. (PDF) [file ppat.1003177.s004.pdf]

Figure S4

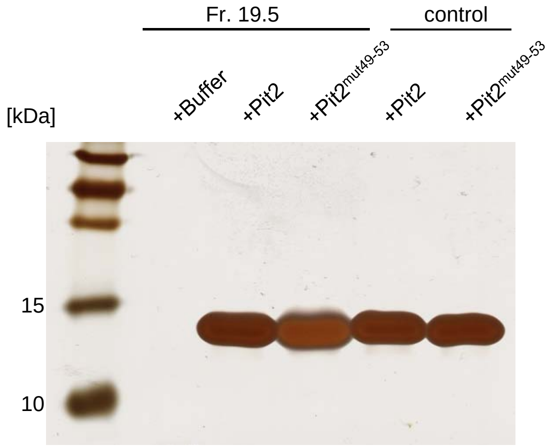

Figure S4: Silver stained SDS-PAGE showing FPLC purified Pit2 and Pit2$^{mut49-53}$. Treatment of both Pit2 versions with apoplastic protease fraction 19.5ml (Fraction 19.5; see Figure 2B) does not generate detectable degradation products.
