# Supplementary material for: Compatibility in the Ustilago maydis–Maize Interaction Requires Inhibition of Host Cysteine Proteases by the Fungal Effector Pit2
Source: PLoS Pathog. 2013 Feb 14;9(2):e1003177. doi: 10.1371/journal.ppat.1003177 (PMC3573112; doi:10.1371/journal.ppat.1003177)
Supplement: Figure S5 — Western Blot analysis to test stability of mCherry-HA tagged Pit2-fusion-proteins expressed by U. maydis during plant infection. Protein extracts of U. maydis infected maize leaves were probed using anti-HA-antibodies. Expected sizes for Pit2-mCherry-HA: 38.8 kDa, Pit2Δ44–57-mCherry-HA: 37 kDa, Pit2mut49–53-mCherry-HA: 38.4 kDa. No signal was detected in samples from maize leaves that were infected with the U. maydis wild type strain SG200. (PDF) [file ppat.1003177.s005.pdf]

Figure S5

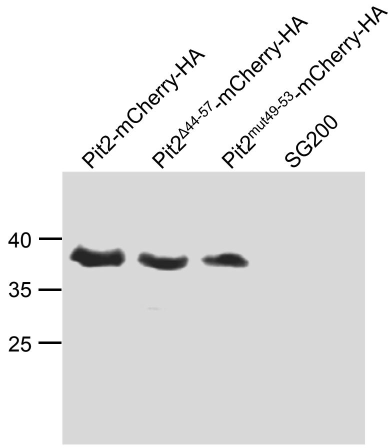

Figure S5: Western Blot analysis to test stability of mCherry-HA tagged Pit2-fusion-proteins expressed by *U. maydis* during plant infection. Protein extracts of *U. maydis* infected maize leaves were probed using anti-HA-antibodies. Expected sizes for Pit2-mCherry-HA: 38.8kDa, Pit2$^{\Delta 44\text{-}57}$-mCherry-HA: 37kDa, Pit2$^{mut49\text{-}53}$-mCherry-HA: 38.4kDa. No signal was detected in samples from maize leaves that were infected with the *U. maydis* wild type strain SG200.
