# Supplementary material for: Compatibility in the Ustilago maydis–Maize Interaction Requires Inhibition of Host Cysteine Proteases by the Fungal Effector Pit2
Source: PLoS Pathog. 2013 Feb 14;9(2):e1003177. doi: 10.1371/journal.ppat.1003177 (PMC3573112; doi:10.1371/journal.ppat.1003177)
Supplement: Table S1 — Oligonucleotides used in this study. (DOCX) [file ppat.1003177.s006.docx]

**Table S1:** Oligonucleotides used in this study

| **Name** | **Sequence (5’🡪3’)** | **Gene accession No. (NCBI)** |  |
| --- | --- | --- | --- |
| OPit2-XhoI-fw | GACTCGAGATTCCGGTGCGTCGATCG | XP_757522 | Cloning of Pit2 and Pit2^mut49-53^ in pRSET-GST-PP |
| OPit2-EcoRI-rv | TTGAATTCTTATTCCCAGATGACCACATCTCCG |  | Cloning of Pit2 and Pit2^mut49-53^ in pRSET-GST-PP, pGBKT7 |
| OPit2^mut49-53^ | CTGGCAAGCTCAACCGGAGAGGAGGAGCCGGCGGCACAGGTTCGCTCGGCAAGGAA |  | Insertion of point mutations in Pit2 |
| OPit2-NcoI-fw | CGTCCATGGTTCCGGTGCGTCGATCGCTCTC |  | Cloning of Pit2 and Pit2^mut49-53^ in pGBKT7 |
| OPit2^Δ44-57^-fw | GGCAAGGAACCTGACAACGG |  | Construction of  pGBKT7-Pit2^Δ44-57^ and P123-Ppit2-pit2^Δ44-57^ |
| OPit2^Δ44-57^-rv | GCCAGCAGCCGAGCTCATTGA |  |  |
| OPit2^Δ49-53^-fw | ACAGGTTCGCTCGGCAAGGAAC |  | Construction of  pGBKT7-Pit2^Δ49-53^ |
| OPit2^Δ49-53^-rv | TCTCCGGTTGAGCTTGCCAG |  |  |
| OPit2-SacII-fw | TATCCGCGGATGCTGTTTCGCTCAGCCTTTG |  | Construction of  P123-Ppit2-pit2^Δ44-57^-mCherry-HA, P123-Ppit2-pit2^mut49-53^-mCherry-HA, P123-Ppit2-pit2-mCherry-HA |
| OmCherryHA-rv | TATGCGGCCGCTTAAGCGTAATCTGGAACATCGTATGGGTACTTGTACAGCTCGTCCATGC |  | Construction of  P123-Ppit2-pit2^Δ44-57^-mCherry-HA, P123-Ppit2-pit2^mut49-53^-mCherry-HA, P123-Ppit2-pit2-mCherry-HA |
| OCP2_full_-NdeI-fw | GGAATTCCATATGCCCAACTCCGGCTTCGCGGACTCC | NP_001105479 | Cloning of CP2 in pGADT7 |
| OCP2_full_-BamHI-rv | CGCGATCCTCATGCGACAATAGGGTAGG |  |  |
| OCP2-NdeI-fw | GACATATGCGTGCGACCCGGCTCGGC |  | Cloning of CP2 in pGADT7 |
| OCP2-BamHI-rv | TGGATCCTCATGCGACAATAGGGTAGG |  |  |
| OCP1A-NdeI-fw | ATACATATGCGCGCCACCTACCTCGGCGCC | NP_001148706 | Cloning of CP1A in pGADT7 |
| OCP1A-BamHI-rv | TATGGATCCTCATGCGCTGCTCTTCATGC |  |  |
| OCP1B-NdeI-fw | ATACATATGCGCGCCACCTACCTCGGCGT | NP_001149658 | Cloning of CP1B in pGADT7 |
| OCP1B-BamHI-rv | TATGGATCCTCATGCGCTGCTCTTCATGCCATCAGCAGC |  |  |
| OCatB-NdeI-fw | GCGCATATGAATGCACTAAGCAATGTTCCT | NP_001150152 | Cloning of CatB in pGADT7 |
| OCatB-BamHI-rv | TGTGGATCCTTAAACTATAGCTCTTCCAACG |  |  |
| OXCP2-NdeI-fw | ATACATATGAAGGCCACCTACCTGGGCCT | NP_001149806 | Cloning of XCP2 in pGADT7 |
| OXCP2-BamHI-rv | TATGGATCCTCAATGGTCCTTGGTCGGGT |  |  |
| OCP2-Mut1 | CAGGGCCACTGTGGATCAGGCTGGACCTTCAGCACTACTG | NP_001105479 | Insertion of point mutations in CP2 |
| OCP2-Mut2 | CTACACCGATGGATGTGAACGGCGCTGTTCTGGCTGTTGGCTAC |  |  |
| OCP2-Mut3 | CTACTGGCTCATCAAGGGCTCATGGGGCGCTGACTG |  |  |
| OCP1A-XbaI-fw | GTATCTAGAATGGCTGCCTCCACCACG | NP_001148706 | Cloning of CP1A in pGreenII 0029 |
| OCP1A-HA-SacI-rv | GTGGAGCTCTTAAGCGTAATCTGGAACATCGTATGGGTATGCGCTGCTCTTCATGCCGT |  |  |
| OCP2-XbaI-fw | ATCTAGAATGGCCCCACGCCGCCTG | NP_001105479 | Cloning of CP2 in pGreenII 0029 |
| OCP2-HA-SacI-rv | GAGCTCTTAAGCGTAATCTGGAACATCGTATGGGTATGCGACAATAGGGTAGGATG |  |  |
| OCatB-XbaI-fw | GCTCTAGAATGGGCGGCGAACTGCTGCT | NP_001150152 | Cloning of CatB in pGreenII 0029 |
| OCatB-HA-SacI-rv | GAGGAGCTCTTAAGCGTAATCTGGAACATCGTATGGGTAAACTATAGCTCTTCCAACGG |  |  |
| OXCP2-XbaI-fw | CTCTAGAATGGCTTGGTCTTGTGCTCG | NP_001149806 | Cloning of XCP2 in pGreenII 0029 |
| OXCP2-HA-SacI-rv | GTGGAGCTCTTAAGCGTAATCTGGAACATCGTATGGGTAATGGTCCTTGGTCGGGTAGGA |  |  |
