# Supplementary material for: Compatibility in the Ustilago maydis–Maize Interaction Requires Inhibition of Host Cysteine Proteases by the Fungal Effector Pit2
Source: PLoS Pathog. 2013 Feb 14;9(2):e1003177. doi: 10.1371/journal.ppat.1003177 (PMC3573112; doi:10.1371/journal.ppat.1003177)
Supplement: Table S2 — U. maydis strains used in this study. (DOCX) [file ppat.1003177.s007.docx]

**Table S2:** *U. maydis* strains used in this study

| **Strains** | **Genotype** | **Reference** |
| --- | --- | --- |
| SG200 | a1mfa2bW2 bE1 | [Kämper et al., 2006](#_ENREF_16) |
| SG200∆pit2 | a1mfa bW2bE1  ∆um01375::hph | [Doehlemann et al., 2011](#_ENREF_8) |
| SG200∆pit2-pit2 | a1mfa2 bW2bE1  ∆um01375::hph ip^r^[Ppit2-pit2]ip^s^ | [Doehlemann et al., 2011](#_ENREF_8) |
| SG200∆pit2-pit2-mCherry | a1mfa2 bW2bE1  ∆um01375::hph ip^r^[Ppit2::pit2:mcherry]ip^s^ | [Doehlemann et al., 2011](#_ENREF_8) |
| SG200∆pit2-pit2^∆44-57^ | a1mfa2 bW2bE1  ∆um01375::hph ip^r^[Ppit2::pit2^∆44-57^]ip^s^ | this study |
| SG200∆pit2-pit2^mut49-53^ | a1mfa2 bW2bE1  ∆um01375::hph ip^r^[Ppit2::pit2^mut49-53^]ip^s^ | this study |
| SG200-pit2^∆44-57^-mCherry | a1mfa2 bW2bE1 ip^r^[Ppit2::pit2^∆44-57^:mcherry]ip^s^ | this study |
| SG200-pit2^mut49-53^-mCherry | a1mfa2 bW2bE1 ip^r^[Ppit2::pit2^mut49-53^:mcherry]ip^s^ | this study |
| SG200-pit2-mCherry-HA | a1mfa2 bW2bE1 ip^r^[Ppit2::pit2:mcherry:HA]ip^s^ | this study |
| SG200-pit2^∆44-57^-mCherry-HA | a1mfa2 bW2bE1 ip^r^[Ppit2::pit2^∆44-57^:mcherry:HA]ip^s^ | this study |
| SG200-pit2^mut49-53^-mCherry-HA | a1mfa2 bW2bE1 ip^r^[Ppit2::pit2^mut49-53^:mcherry:HA]ip^s^ | this study |
